# Development of a Novel Prognostic Model of Glioblastoma Based on m6A-Associated Immune Genes and Identification of a New Biomarker

**DOI:** 10.3389/fonc.2022.868415

**Published:** 2022-07-20

**Authors:** Na Luo, Xizi Sun, Shengling Ma, Xiaoyu Li, Wenjun Zhu, Min Fu, Feng Yang, Ziqi Chen, Qianxia Li, Yuanyuan Zhang, Xiaohong Peng, Guangyuan Hu

**Affiliations:** ^1^ Department of Oncology, Tongji Hospital, Tongji Medical College, Huazhong University of Science and Technology, Wuhan, China; ^2^ Department of Radiology, Tongji Hospital, Tongji Medical College, Huazhong University of Science and Technology, Wuhan, China; ^3^ Department of Medical Oncology, The First Affiliated Hospital, College of Medicine, Zhejiang, China

**Keywords:** glioblastoma, TCGA, CGGA, m6A-associated immune genes, stratification methods, tumor microenevironment, biomarker

## Abstract

**Background:**

Accumulating evidence shows that m6A regulates oncogene and tumor suppressor gene expression, thus playing a dual role in cancer. Likewise, there is a close relationship between the immune system and tumor development and progression. However, for glioblastoma, m6A-associated immunological markers remain to be identified.

**Methods:**

We obtained gene expression, mutation, and clinical data on glioblastoma from The Cancer Genome Atlas and Chinese Glioma Genome Atlas databases. Next, we performed univariate COX–least absolute shrinkage and selection operator (LASSO)–multivariate COX regression analyses to establish a prognostic gene signature and develop a corresponding dynamic nomogram application. We then carried out a clustering analysis twice to categorize all samples according to their m6A-regulating and m6A-associated immune gene expression levels (high, medium, and low) and calculated their m6A score. Finally, we performed quantitative reverse transcription-polymerase chain reaction, cell counting kit-8, cell stemness detection, cell migration, and apoptosis detection *in vitro* assays to determine the biological role of CD81 in glioblastoma cells.

**Results:**

Our glioblastoma risk score model had extremely high prediction efficacy, with the area under the receiver operating characteristic curve reaching 0.9. The web version of the dynamic nomogram application allows rapid and accurate calculation of patients’ survival odds. Survival curves and Sankey diagrams indicated that the high-m6A score group corresponded to the groups expressing medium and low m6A-regulating gene levels and high m6A-associated prognostic immune gene levels. Moreover, these groups displayed lower survival rates and higher immune infiltration. Based on the gene set enrichment analysis, the pathophysiological mechanism may be related to the activation of the immunosuppressive function and related signaling pathways. Moreover, the risk score model allowed us to perform immunotherapy benefit assessment. Finally, silencing CD81 *in vitro* significantly suppressed proliferation, stemness, and migration and facilitated apoptosis in glioblastoma cells.

**Conclusion:**

We developed an accurate and efficient prognostic model. Furthermore, the correlation analysis of different stratification methods with tumor microenvironment provided a basis for further pathophysiological mechanism exploration. Finally, CD81 may serve as a diagnostic and prognostic biomarker in glioblastoma.

## Introduction

According to the WHO classification of tumors of the central nervous system, WHO grade IV refers to mitotically active, necrosis-prone, cytologically malignant tumors typically related to rapid pre- and post-operative disease evolution and a fatal outcome. Moreover, both isocitrate dehydrogenase (IDH)-wild type and IDH-mutant glioblastoma are grade IV tumors (1–3). Despite continuous progress in glioblastoma treatment research, the multimodal treatment paradigm has remained unchanged for 15 years. It consists of a combination of maximal surgical resection, radiation, and chemotherapy with temozolomide (4). Compared with other solid tumors, biological factors such as the blood–brain barrier, significant molecular heterogeneity, or unique tumor and immune microenvironment make it challenging to develop new therapeutic methods (5), resulting in a low 5-year survival rate for glioblastoma patients (under 10%) (4). Therefore, developing refined stratification methods and identifying tumor biomarkers associated with the factors mentioned above will improve glioblastoma treatment and prognosis.

RNA methyltransferases (“writers”), RNA demethylase (“erasers”), and N^6^-methyladenine (m6A) binding protein (“readers”) regulate the m6A mRNA modification. This process emerged as a glioblastoma research hotspot in the last 4 years (6). Existing studies focused on the role that the m6A modification plays in glioblastoma stem cells (GSCs), which are considered the initiating factor of glioblastoma and the culprit mediating glioblastoma recurrence (7). Some studies suggested that the overexpression of m6A “writers” (such as METTL3 or METTL14) (8) or the inhibition of m6A “erasers” [such as FTO (9) or ALKBH5 (10)] inhibited self-renewal and tumorigenesis of GSCs. Meanwhile, other studies observed elevated METTL3 expression (11) and imply that its methylation activity plays an oncogenic role (12) in GSCs. Moreover, a study by Dixit et al. (13) showed that the “reader” YTHDF2 maintained the expression of the oncogene MYC in GSCs only and was essential for GSC maintenance. This contradiction calls for in-depth studies of the mechanism of m6A regulators on glioblastoma cell tumorigenesis and invasiveness to identify therapeutic targets.

Although studies on immunotherapy against glioblastoma have included various approaches (immune checkpoint blockade, vaccine therapies, chimeric antigen receptor T-cell therapies, and oncolytic viral therapies), none have shown a definite effect in phase 3 trials (4). The main reason for this situation lies in the local and systemic immunosuppression observed in glioblastoma patients, and the mechanism underlying the latter remains largely unknown (14). Regarding local immunosuppression, we should first note that glioblastoma is a highly vascularized malignant tumor with dense tortuous and leaky blood vessels, allowing many immune cells to infiltrate the tumor core. Cells infiltrating the tumor include microglia-derived and bone marrow-derived tumor-associated macrophages, microglia, and T cells (15). The immune microenvironment mainly mediates three immunosuppression aspects: changes in glioblastoma cell surface molecules inhibit the immune response (16–18); the glioblastoma microenvironment is rich in immunosuppression-mediating factors, including transforming growth factor β (TGF-β) (19), interleukin 10 (IL-10) (20), prostaglandin E-2 (PGE2) (21), colony-stimulating factor 1 (CSF-1) (22), vascular endothelial growth factor (VEGF) (23), arginase 1 (24), indoleamine 2,3-dioxygenase (IDO) (25), macrophage migration inhibitory factor (MIF) (26), and interleukin-6 (IL-6) (27); immunosuppressive cells such as regulatory T cells (Tregs) (28), tumor-associated macrophages (29), and monocytes with an immunosuppressive phenotype (14) are over-represented in the glioblastoma microenvironment. It is worth mentioning that the medium-level mutational burden of glioblastoma implies that the lack of a defining mutation hinders the development of targeted therapy and immunotherapy (30).

Growing evidence suggests that immune cells require m6A for many processes, including development, differentiation, activation, migration, and polarization (31, 32). Take the role of m6A modification in T cells as an example. Studies indicated that m6A modification regulated naive T-cell differentiation (33) and maintained the suppressor function of Tregs (34) by specifically targeting SOCS (suppressor of cytokine signaling) family genes in different T-cell subtypes. Furthermore, the m6A modification promoted T follicular helper cell differentiation programs in a METTL3-dependent manner by increasing the stability of transcription factor 7 transcripts (35). Immunotherapy is a new cancer therapy approach that stimulates and improves the immune system’s natural ability to fight cancer cells (36). However, it comes with two main challenges (1): finding a strategy to improve efficacy and (2) figuring out biomarkers to predict the outcomes (37). The role of m6A regulators in these challenges recently became a research hotspot. Yang et al. (38) observed that knocking down FTO sensitized melanoma cells to interferon-gamma (IFN-γ) and sensitized melanoma to anti-PD-1 treatment in mice. In another study (39), depleting FTO decreased PD-L1 expression in colon cancer cells. Li et al. (40) found that ALKBH5 loss or inhibition enhanced the response to anti-PD-1 therapy in melanoma and colorectal cancer. Moreover, YTHDF1 can control anti-tumor immunity and improve the efficacy of PD-L1 checkpoint blockade by regulating lysosomal proteases expression in an m6A-dependent manner (41).

After downloading multiple public datasets, we built a prognostic model of m6A-associated immune genes using univariate COX–least absolute shrinkage and selection operator (LASSO)–multivariate COX regression analyses and developed a dynamic nomogram web application. We introduced and assessed three other stratification methods, including two types of clustering and an m6A score. Additionally, an enrichment analysis revealed potential immunosuppressive mechanisms. Next, we performed stemness and tumor microenvironment (TME) correlation, copy number variation (CNV), and mutational analysis on risk model genes. We also conducted immunotherapy-efficacy prediction in different risk groups. Finally, *in vitro* assays revealed the biological role of CD81 in glioblastoma cells.

## Methods

### Data Acquisition and Differential Analysis

Wu and Bai (42) [PMID: 34686691] identified 23 m6A regulators and inspired us for this study. We acquired gene expression data (HTSeq–FPKM), clinical information, and glioblastoma mutation data from the data portal of The Cancer Genome Atlas (TCGA). We also downloaded the mRNAseq_325 and mRNAseq_693 datasets, and corresponding clinical data for external validation from the Chinese Glioma Genome Atlas (CGGA). We extracted immune-related genes from the Gene Set Enrichment Analysis (GSEA) website. We pre-treated all the data from diagnosed patients with the limma and sva packages.

We first excavated prognostic genes in m6A-regulating genes *via* univariate COX and Kaplan–Meier analysis, followed by coexpression analysis of m6A-regulating genes by running the psych package. Next, we calculated the optimized cutoff value with the surv_cutpoint function in the survival package to get high- and low-expression groups for each gene. We then calculated the correlation coefficient between m6A-regulating and immune-related genes using wilcox.test and obtained m6A-associated immune genes for further analysis (|correlation coefficient| > 0.6 and *p* < 0.05). Based on these data, we visualized coexpression networks of m6A-regulating genes on the one hand and m6A-regulating genes and m6A-associated immune genes on the other using the igraph package. Lastly, we identified differentially expressed m6A-associated immune genes between normal and tumor samples using the limma package [false discovery rate (FDR) < 0.05 and |logFC| > 0.5].

### Prognostic Model Development, Mutation Analysis, and Dynamic Nomogram Construction

We randomly split the glioblastoma sample data from TCGA into a training group and an internal validation group and used CGGA samples as an external validation group. For the training group, we used classical univariate COX–LASSO algorithm–multivariate COX stepwise regression to get prognostic genes (*p* < 0.05), exclude high correlation genes, and finally construct an optimized prognostic model. We divided the sample data into high-risk and low-risk groups using the training group median risk score as the cutoff value. Next, we plotted the survival and receiver operating characteristic (ROC) curves to assess the predictive power of the model.

After building the prognostic model, we visualized gene-mutation waterfall plots of the high- and low-risk groups using the maftools software package. Next, we calculated the impact of mutation burden on survival in the high- and low-risk groups.

To enhance the translational significance of our prognostic model, we loaded the DynNom package to develop a corresponding web version of the dynamic-nomogram application allowing rapid and accurate patient prognostic calculation.

### Clustering and Tumor Microenvironment Analysis

We performed a clustering analysis based on m6A-regulating genes with the ConsensusClusterPlus package on all samples (Kmax = 9). We calculated the TME score of each sample *via* the estimate package in R. For each sample, we analyzed 23 immune cell content by conducting a single sample GSEA analysis using the GSVA package. We plotted heatmap and violin plots of TME scores along with survival curves to visualize the correlation between m6A and TME, especially in different immune cells.

Likewise, we performed a clustering analysis with ConsensusClusterPlus on all samples based on m6A-associated prognostic immune genes. We also produced heatmap and violin plots of TME scores along with survival curves according to the scoring results. Furthermore, we obtained GSEA enrichment curves using the org.Hs.eg.db R package. These plots show the top five Gene Ontology (GO) terms and Kyoto Encyclopedia of Genes and Genomes (KEGG) pathways, which were significantly enriched in the high-expression group (*p* < 0.05).

### m6A Score Calculation and Sankey Diagram Plotting

Using the limma package, we identified the genes expressed differentially in the high, medium, and low m6A regulating gene expression groups. We obtained the final list of differentially expressed genes by loading the VennDiagram package and taking the intersection of differentially expressed genes between the groups above. Next, we screened prognostic genes by applying the univariate COX method (*p* < 0.05), according to which the m6A score of each sample was calculated by the principal component analysis (PCA) method. Based on the m6A score of each sample, we calculated the optimized cutoff value with the surv_cutpoint function. We then separated the samples into high and low m6A score groups and drew their corresponding survival curves.

To directly display the corresponding relations among the m6A cluster, immune-gene cluster, m6A score, and risk score, we plotted a Sankey diagram using the ggalluvial package. Moreover, we visualized the correlation between the m6A score and the two clustering types through a box plot. Additionally, we built a correlation matrix of the m6A score and predicted immune cell content in TME with the corrplot package.

### Tumor Microenvironment, Copy Number Variation, and Mutation Analysis of Model Genes

Next, we calculated the correlation of each model gene with stemness and TME scores. We downloaded stemness scores based on DNA methylation and RNA from UCSC Xena and calculated TME scores with the estimate package. At the same time, based on the Tumor Immune Estimation Resource (TIMER) database, we calculated and analyzed both the CNV frequency of model genes and the correlation between model gene CNV and immune cell infiltration level. Furthermore, we downloaded data about mutation frequency and domain mutations of model genes from the cBioportal database.

### Benefit Evaluation of Immunotherapy and Model Comparison

To figure out whether the prognostic model could be used to evaluate immunotherapy efficacy, we analyzed a series of immunotherapy biomarkers based on the TCGA glioblastoma dataset. We uploaded TCGA sample expression profiles to the tumor immune dysfunction and exclusion (TIDE) database [PMID: 30127393] to obtain the TIDE, microsatellite instability (MSI), and dysfunction and exclusion scores of each sample. We then compared these scores between different risk groups.

Next, we constructed a 3-year ROC curve to compare the prognostic prediction power of the risk, TIDE, and tumor inflammation signature (TIS) scores [PMID: 29929551].

### Cell Lines and siRNA Transfection

We purchased glioblastoma cells U87, U251, and U118 and human astrocyte cell line HA1800 from the American Type Culture Collection (ATCC) and cultured them in Dulbecco’s modified Eagle medium (DMEM) (HyClone, USA) with 10% fetal bovine serum (Gibco, USA) at 37°C in 5% CO_2_. We purchased 100 nM siRNA from RIBO Biotechnology and transfected the cells with it by incubating them for 24–72 h using HighGene Transfection reagent (Abclonal, China).

### Western Blotting and qRT-PCR

We used RIPA lysis buffer containing phenylmethanesulfonyl fluoride to collect proteins from U251 and U118 cells. We then separated the proteins by 10% SDS-PAGE and transferred them to a 0.45-µm polyvinylidene fluoride membrane (Millipore, USA). Next, we blocked the membrane with 5% skim milk at room temperature for 1 h and performed immunoblotting using the antibodies indicated hereafter, followed by an enhanced chemiluminescence detection kit (Thermo Fisher Scientific). We used the following antibodies: CD81 mouse monoclonal antibody, 1:000(Proteintech 66866-1-lg);SOX10 rabbit monoclonal antibody, 1:1000 (Abclonal A8655);.nanog rabbit polyclonal antibody, 1:1000 (Proteintech 14295-1-Ap).

We extracted RNA from U87, U251, and U118 cells using si-CD81 and negative control (NC) siRNA as a control. Then, we converted the obtained RNA into cDNA using real-time PCR with a SYBR Green qPCR mix (Vazyme, China) and the following primers:

CD81-Forward: TTCCACGAGACGCTTGACTG;
CD81-Reverse: CCCGAGGGACACAAATTGTTC;
GAPDH-Forward: GACCACAGTCCATGCCATCA;
GAPDH-Reverse: GTCAAAGGTGGAGGAGTGGG.

### Cell proliferation and Apoptosis Assays

We assessed proliferation and apoptosis using a CCK-8 assay and flow cytometry, respectively. After transfection with CD81 siRNA for 48 h, we cultured U251 and U118 cells in 96-well plates (2,000 cells/plate in 100 µl of DMEM). We monitored the proliferative capacity of treated cells at 24, 48, 72, and 96 h. We added the CCK8 reagent (Yeasen, Shanghai, China) to each plate according to the manufacturer’s instructions and obtained the OD_450_ value with a microplate reader (BioTek, USA).

### Migration and Invasion Assays

We analyzed cellular migration through transwell and wound healing assays. For the transwell migration assay, we inoculated 40,000 cells into the upper chambers using Matrigel-coated transwell inserts (BD Biosciences, USA). After 20 h of incubation, we washed the insert plates three times with 1 × phosphate-buffered solution. We then fixed the cells beneath the membrane with 95% ethyl alcohol and stained them with 1% crystal violet for 20 min at room temperature. Finally, we counted the cells that passed through the membrane with a microscope. For the wound healing assay, we cultured U251 and U118 cells in 6-well plates and scraped them with a 200-μl pipette tip. Then, we cultured the cells in DMEM without fetal bovine serum, captured images of the wounds at 0, 12, and 24 h, and quantified the area of the wounds using ImageJ software.

### Statistical Analysis

Except for open database and special software, we performed all general statistical analysis and graph plotting of bioinformatic analysis using the R programming language (Version 4.0.3). We compared the groups using two-tailed Student’s *t*-tests. We constructed the optimal prognostic model using classical univariate COX–LASSO algorithm–multivariate COX stepwise regression analysis. We considered *p* < 0.05 as statistically significant.

## Results

### Identification of Prognostic Genes and Construction of Coexpression Networks

**Figure 1:**
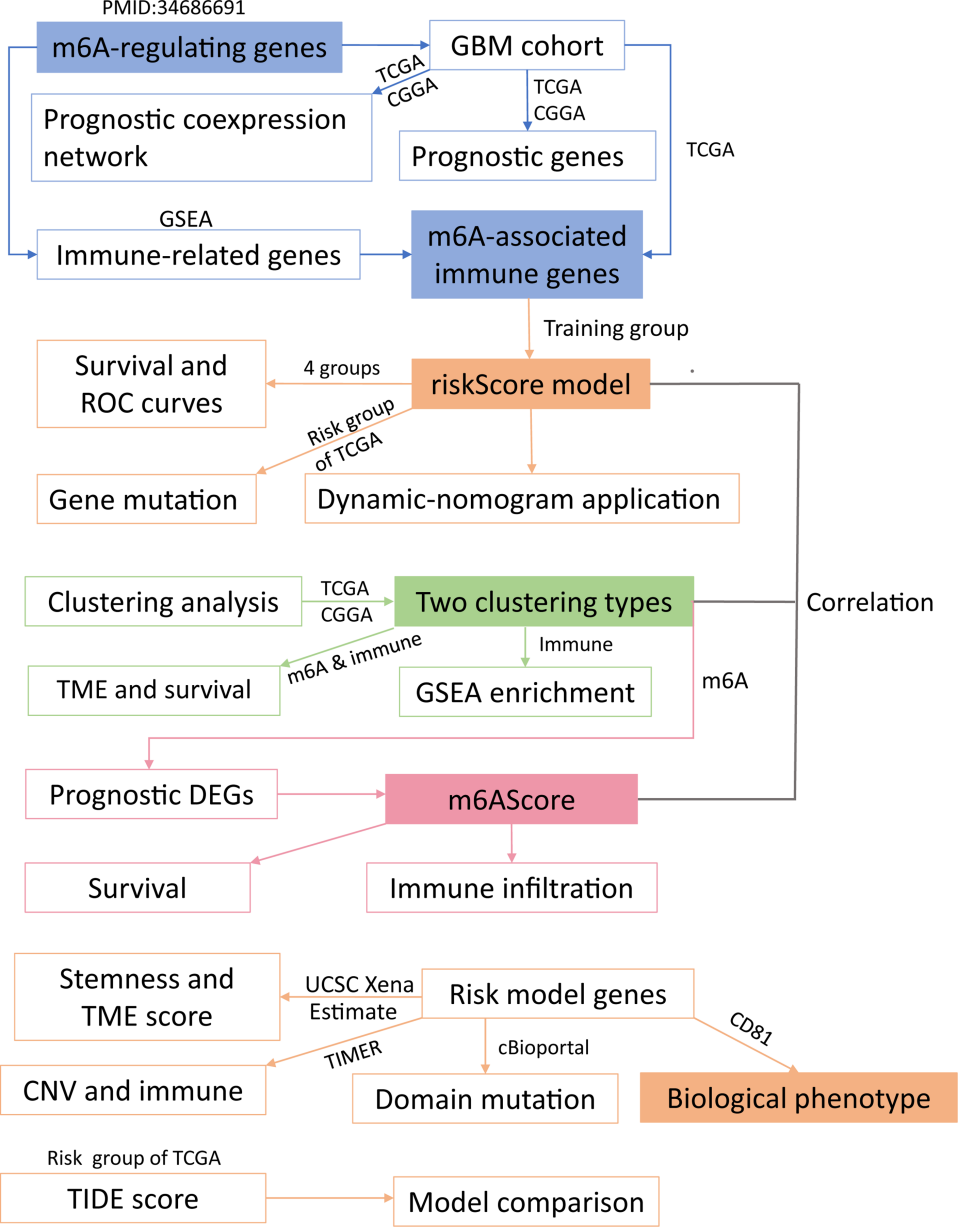
Workflow of this study. The whole study could be split into five parts (modules with different background colors) (1): identification of m6A-associated immune genes (2); construction of a risk score model (3); sample clustering in two ways (4); study of the m6A score; and (5) experimental validation. Overall, we grouped glioblastoma patients using four grouping methods and explored correlations among the obtained groups. The detailed analysis of these five parts is presented in the same color as the core module.

**Figure 2:**
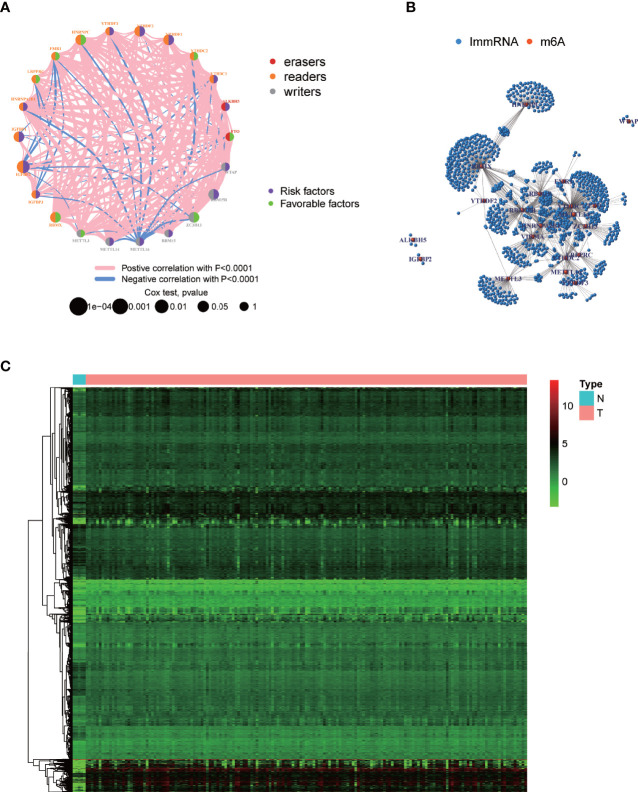
Visualization of m6A-regulating genes and m6A-associated immune genes. **(A)** m6A prognostic network diagram by running the psych package. The nodes represent m6A-regulating genes. Red and blue lines respectively indicate positive and negative coexpression relationships. The different colors of the circles indicate the different characters of m6A-regulating genes. The size of the circles indicates the *p*-values. **(B)** Coexpression network between m6A-regulating and m6A-associated immune genes based on wilcox.test. Red nodes represent m6A-regulating genes, blue nodes represent m6A-associated immune genes, and the connections represent coexpression relationships. **(C)** Heatmap of differentially expressed m6A-associated immune genes by running limma package. N, normal group; T, tumor group. Green, black, and red indicate low, medium, and high expression levels, respectively.

**Figure 1** displays the study workflow. **Table 1** lists the m6A regulators analyzed as m6A-regulating genes in this study. A total of 23 m6A regulators, namely, 13 “readers”, 8 “writers”, and 2 “erasers”, were identified. All TCGA-GBM samples belong to grade IV, and we collated their overall clinical information in **Table 2**. Furthermore, we carried out survival analysis in TCGA and CGGA samples and recorded the results in **Supplementary File 1**. We found that there were 8 m6A-regulating genes with *p* < 0.05 in the Kaplan–Meier survival analysis (**Supplementary Figure 1**). Among these genes, HNRNPC, RBMX, and ZC3H13 were high-risk prognostic factors, while IGFBP1, IGFBP2, RBM15B, YTHDF1, and YTHDF2 were low-risk prognostic factors. We then explored the coexpression relationship among m6A-regulating genes and immune genes. The prognostic coexpression network explicates the coexpression relations among m6A-regulating genes, which mainly consist of positive correlations (**Figure 2A**). It also displays the *p*-values of the univariate COX regression analysis and whether the genes are risk factors or favorable genes. Likewise, the coexpression network of m6A-regulating genes and m6A-associated immune genes (|correlation coefficient| > 0.6) indicates that HNRNPC, RBM15B, RBMX, and ZC3H13 are coexpressed with relatively more immune genes than other m6A-regulating genes (**Figure 2B**). Lastly, differential analysis result is visualized in the heatmap (**Figure 2C**), which shows the differentially expressed m6A-associated immune genes (FDR < 0.05 and |logFC| > 0.5) between the normal and tumor groups.

**Table 1:** List of N^6^-methyladenine (m6A) regulators.

Regulator	Category	Regulator	Category	Regulator	Category
METTL3	Writer	YTHDC1	Reader	HNRNPA2B1	Reader
METTL14	Writer	YTHDC2	Reader	IGFBP1	Reader
METTL16	Writer	YTHDF1	Reader	IGFBP2	Reader
WTAP	Writer	YTHDF2	Reader	IGFBP3	Reader
VIRMA	Writer	YTHDF3	Reader	RBMX	Reader
ZC3H13	Writer	HNRNPC	Reader	FTO	Eraser
RBM15	Writer	FMR1	Reader	ALKBH5	Eraser
RBM15B	Writer	LRPPRC	Reader		

The corresponding genes were recognized as m6A-regulating genes in this study.

**Table 2:** Clinical characteristics of glioblastoma patients in the TCGA database.

Characteristics	Total Patients (*N* = 594)	
	*N*	%
Age (years)		
<60 years	307	51.68
≥60 years	287	48.32
Gender		
Female	230	38.72
Male	364	61.28
Grade		
I	0	0.00
II	0	0.00
III	0	0.00
IV	594	100.00
Survival status		
Alive	144	24.24
Dead	450	75.76
P/R		
Primary	543	91.41
Recurrent	20	3.37
Unknown	31	5.22

### Construction of the Prognostic Model and Corresponding Dynamic Nomogram Along With the Mutation Analysis Results

**Figure 3:**
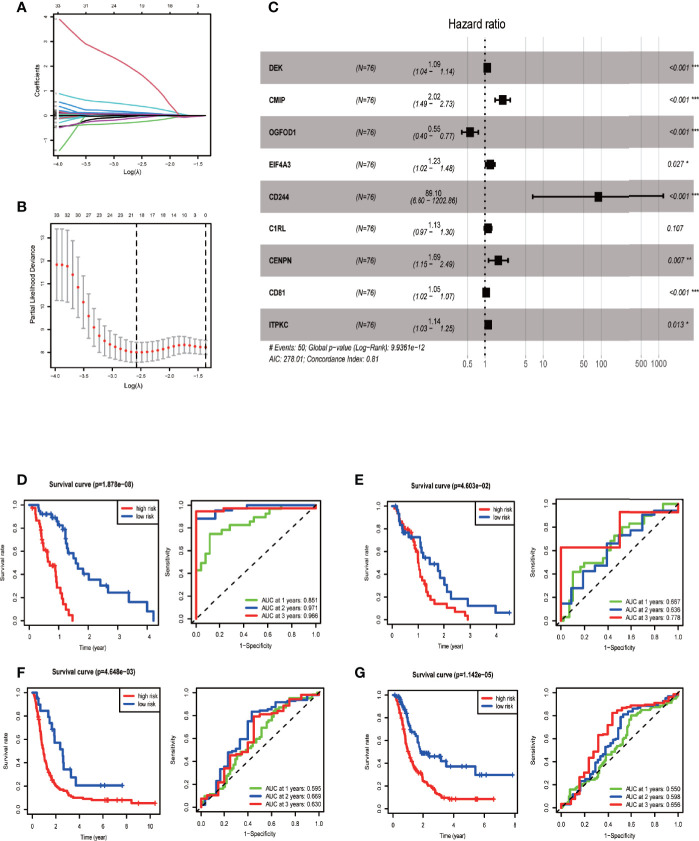
Establishing and evaluating the prognostic model. **(A)** LASSO coefficient plot. LASSO coefficient spectrum of 59 genes screened by univariate COX analysis in glioblastoma. Coefficient distribution map for a logarithmic (λ) sequence. **(B)** λ selection figure. Selecting the best parameters for glioblastoma in the LASSO model (λ) of the training group. **(C)** Forest map based on multivariate COX regression showing HR values and 95% confidence intervals for all model genes. **(D)** Model evaluation *via* survival and ROC curves in the training group. **(E)** Model evaluation in the internal validation group. **(F)** Model evaluation in one external validation group (CGGA mRNAseq_325 dataset). **(G)** Model evaluation in another external validation group (CGGA mRNAseq_693 dataset).

We provided the specific grouping of the training (**Supplementary File 2**), internal validation (**Supplementary File 3**), and external validation groups (**Supplementary Files 4, 5**) as supplementary files. Moreover, we constructed a risk model of m6A-associated immune genes based on the training group. The univariate COX analysis identified 59 prognostic m6A-associated immune genes (**Supplementary File 6**). Following the elimination of high correlation genes by the LASSO algorithm (**Figures 3A, B**), we then constructed a prognostic model *via* multivariate COX stepwise regression as follows (**Figure 3C** and **Table 3**):


$$\begin{array}{l} \text{risk score of each patient}=0.088\times DEK+0.702\times CMIP+∼(-0.593)\times OGFOD1+0.207\\ \times EIF4A3+4.490\times CD244+0.119\times C1RL+0.527\times CENPN\\ +0.045\times CD81+0.127\times ITPKC \end{array}$$

**Table 3:** List of the model genes.

id	coef	HR	HR.95L	HR.95H	*p*-value
DEK	0.087748	1.091713	1.042578	1.143164	0.000188
CMIP	0.701543	2.016863	1.492289	2.725838	5.00E-06
OGFOD1	−0.59329	0.552509	0.398074	0.766858	0.00039
EIF4A3	0.206906	1.229867	1.02439	1.47656	0.026533
CD244	4.489779	89.10175	6.600188	1202.863	0.000722
C1RL	0.118827	1.126175	0.974588	1.301339	0.107181
CENPN	0.527471	1.694641	1.151905	2.493095	0.007408
CD81	0.045468	1.046517	1.023563	1.069986	5.87E-05
ITPKC	0.126808	1.135199	1.027309	1.254419	0.012819

The univariate and multivariate COX regression analyses indicated that risk score was a prognostic factor independent of age, sex, IDH mutation status, and G-CIMP carrier status (**Supplementary Figure 2**).

We then tested the performance of the model in training and validation sets. According to the median risk score of the training group, we divided samples of the training, internal validation, and external validation groups into high-risk and low-risk groups. Survival curves showed that the high-risk group had a significantly lower survival rate than the low-risk group for each analyzed sample group. The area under the ROC curve of the training group was 0.8–0.9, and that of each validation group was around 0.6, indicating the relatively high predictive power of our prognostic model (**Figures 3D–G**).

Additionally, we explored gene mutation in different risk groups and found that the genes with the highest mutation frequency in the high-risk group were PTEN, TP53, and TTN (**Supplementary Figure 3A**). In contrast, those in the low-risk group were PTEN, TP53, and EGFR (**Supplementary Figure 3B**). Furthermore, we compared survival among different groups and learned that survival curves did not show apparent difference in prognosis between groups with a different mutation burden. The result indicated that risk score determined prognosis relatively independent of mutation burden (**Supplementary Figure 3C**).

The web version of the dynamic nomogram application we built (URL: https://u20131050.shinyapps.io/GBM-m6A_ImmRNA-Dynamic_nomogram/) calculates patient survival rate online directly by adjusting the expression level of each model gene.

### Two Sample Clustering Methods and their Relationships with the Tumor Microenvironment

**Figure 4:**
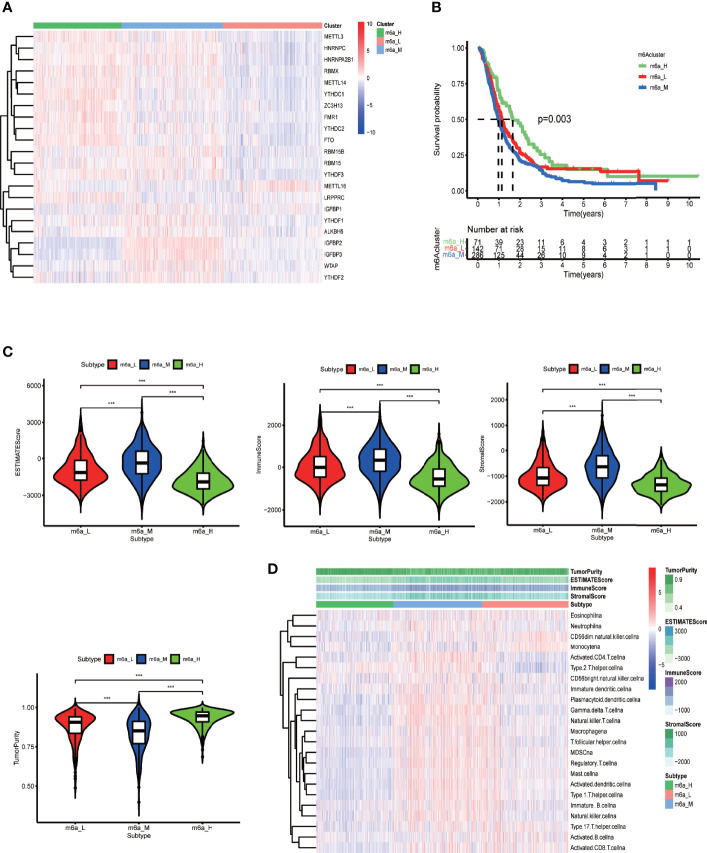
Clustering based on m6A-regulating genes and TME scores of different clusters. **(A)** Clustering heat map of TCGA and CGGA samples by running the ConsensusClusterPlus package. **(B)** Survival curves of different clusters based on the survival package. **(C)** Violin plots of TME scores of different clusters. The TME scores include ESTIMATE score, immune score, stromal score, and tumor purity. **(D)** Heat map of TME and specific immune cell content in different clusters obtained by ssGSEA analysis. The abscissa represents the sample name, and the ordinate represents TME scores and different immune cells. ***p < 0.001, **p < 0.01 *p < 0.05, ns: *p* > 0.05.

After running ConsensusClusterPlus package in R, we obtained an appropriate increase of the area under the cumulative distribution function curve and tight intra-group linkage when K = 3 (**Supplementary Figure 4A**). We therefore grouped the TCGA and CGGA samples into three clusters based on their m6A-regulating gene expression levels (high, medium, or low), as shown in the heatmap (**Figure 4A**). The survival analysis revealed that the medium- and low-expression groups had lower survival rates than the high-expression group (*p* = 0.03) (**Figure 4B**). In addition, TME scoring results indicated that the medium- and low-expression groups had higher immune cell and stromal cell contents—and thus lower tumor purity—than the high-expression group. Additionally, the medium-expression group had the lowest tumor purity and the poorest prognosis (**Figures 4B–D**).

**Figure 5:**
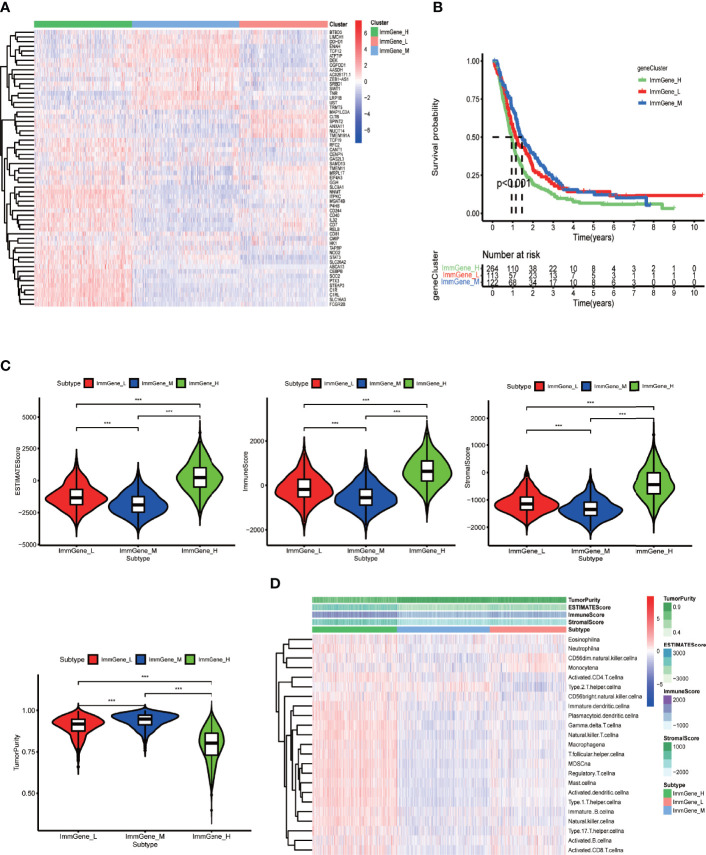
Clustering based on prognostic m6A-associated immune genes and TME scores of different clusters. **(A)** Clustering heat map of TCGA and CGGA samples by running the ConsensusClusterPlus package. **(B)** Survival curves of different clusters based on survival package. **(C)** Violin plots of TME scores of different clusters. The TME scores include ESTIMATE score, immune score, stromal score, and tumor purity. **(D)** Heat map of TME scores and specific immune cell content in different clusters obtained by ssGSEA analysis. The abscissa represents the sample name, and the ordinate represents TME scores and different immune cells. ***p < 0.001, **p < 0.01 *p < 0.05, ns: *p* > 0.05.

**Figure 6:**
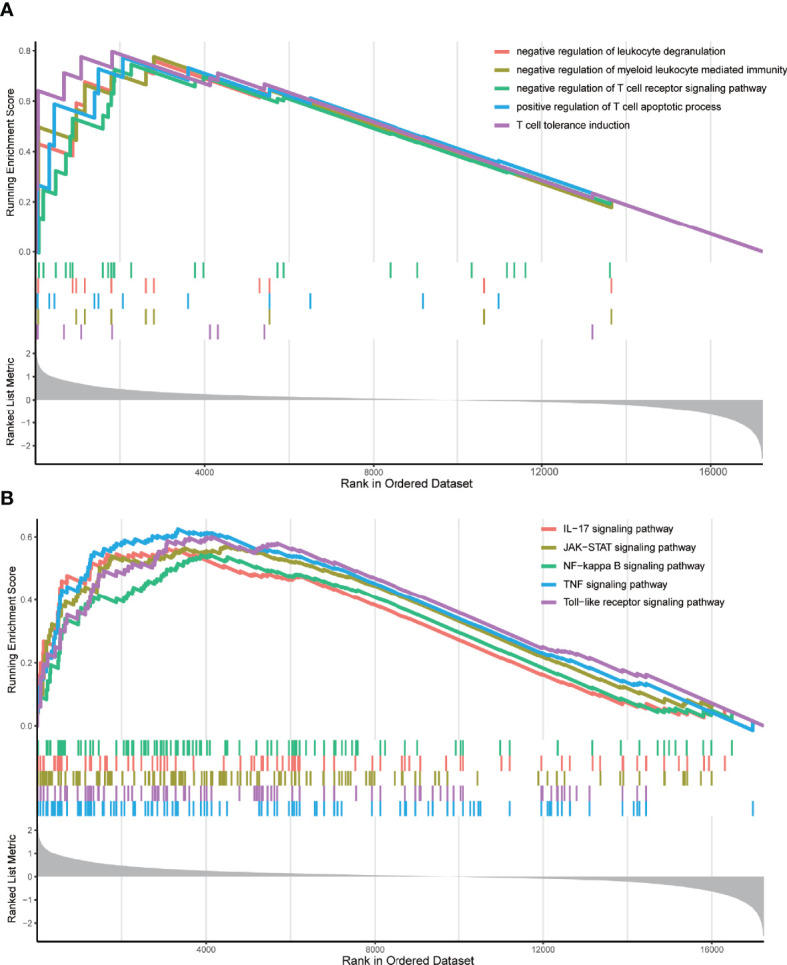
GSEA enrichment analysis. **(A)** The top five GO enrichment results of the enrichment score. These GO terms were prominently enriched in the group with high m6A-associated immune gene expression levels. **(B)** The top five KEGG enrichment results of the enrichment score. These KEGG pathways were prominently enriched in the group with high m6A-associated immune gene expression levels.

Next, for the same reason as above, we grouped the TCGA samples into 3 clusters (**Supplementary Figure 4B**) according to their prognostic m6A-associated immune gene (**Supplementary File 6**) expression (high, medium, or low) (**Figure 5A**). The survival analysis revealed that the high-expression group had a lower survival rate than the other groups (**Figure 5B**). We noticed that the TME analysis revealed the highest immune infiltration and the lowest tumor purity in the high-expression group, which was in complete contrast to the m6A-regulated gene clustering analysis (**Figures 5C, D**). Moreover, GSEA enrichment analysis showed that high prognostic m6A-associated immune gene expression groups were enriched in negative regulation of leukocyte degranulation, myeloid leukocyte-mediated immunity, and T-cell receptor signaling pathway. These results suggested an interesting phenomenon: activating the immunosuppressive function in patients expressing high prognostic m6A-associated immune genes levels could lower the survival rate (**Figure 6**). **Supplementary File 7** contains the GSEA results of the top five GO and KEGG items of enrichment score.

### Clustering Based on m6A Score and Relationships Between the Different Grouping Methods

**Figure 7:**
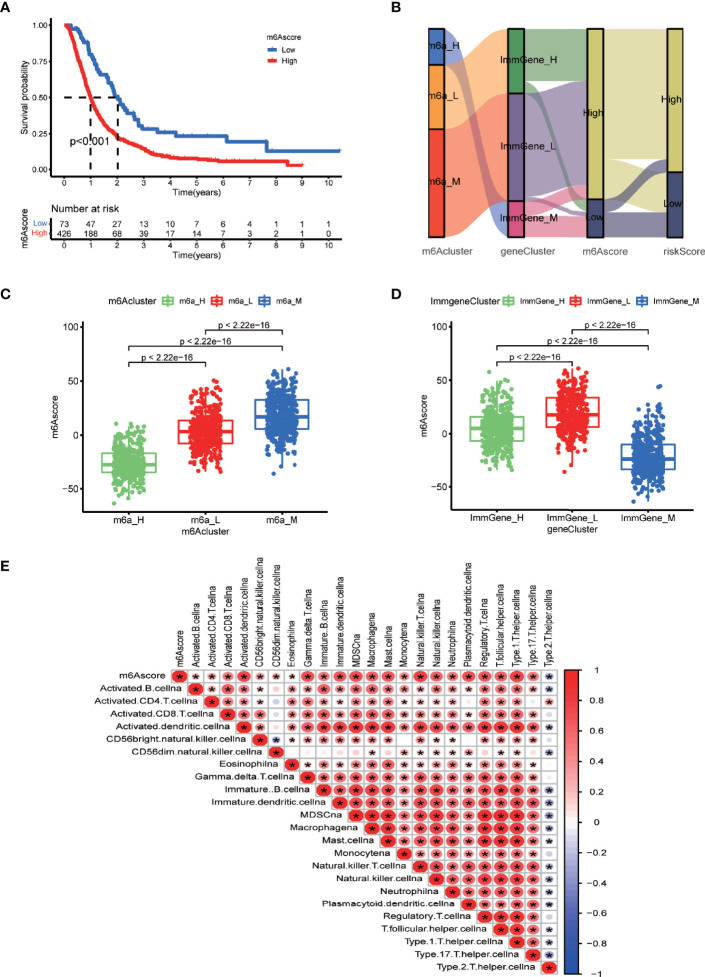
m6A scores and correlations between the different grouping methods. **(A)** Survival analysis of m6A score groups by running survival package in R. **(B)** Sankey diagram displaying the correlations between m6A score, the other two clustering types, and risk score. **(C)** Boxplot comparing m6A scores among the different m6A-regulating gene clusters. **(D)** Boxplot comparing the m6A scores among the different m6A-associated immune gene clusters. **(E)** Correlation matrix between m6A scores and immune cell infiltration *via* the corrplot package. Red indicates a positive correlation, and blue indicates a negative correlation. Asterisks indicate significant correlations.

We then explored a fourth way of grouping glioblastoma patients. **Supplementary File 8** contains the m6A score of each sample as a table. Survival analysis indicated that the high-m6A score group had a significantly lower survival rate than the low-m6A score group (**Figure 7A**). Furthermore, we found the correspondence among the four ways of stratification. The Sankey diagram (**Figure 7B**) and boxplots (**Figures 7C, D**) revealed an important trend: the high-m6A score group corresponded to the medium and low m6A-regulating gene expression groups and the high m6A-associated prognostic immune gene expression group. Furthermore, we analyzed the correlation between m6A score and the content of various immune cells in the TME. The result clearly showed significant positive correlations with predicted content of all analyzed immune cells in the TME, except for type 2 T helper cells (**Figure 7E**), which suggested higher immune infiltration in the high-m6A score group. Thus, medium m6A-regulating gene expression, high prognostic m6A-associated immune gene expression, high-m6A score, and high-risk score all indicated lower survival rates. Furthermore, there was correspondence among these clusters to some extent.

### Multi-Omics Analysis of Model Genes and the Relationships Between Copy Number Variation and Immune Cell Iinfiltration

Based on the prognostic model above, we next evaluated the relationships of each model gene to stemness and TME score. We preliminarily estimated that DEK, CD244, C1RL, CD81, and ITPKC were related to stemness and TME scores (*p* < 0.05) (**Supplementary Figure 5**).

CNV and mutation frequency analysis showed that the vast majority of model genes had CNV frequencies lower than 3% and mutation frequencies lower than 1%, suggesting that both the CNV frequency (**Supplementary Figure 6A**) and mutation frequency (**Supplementary Figure 6B**) of model genes were rather low. Furthermore, the CNV of all model genes, except C1RL, could influence certain immune cell infiltration levels in glioblastoma (**Supplementary Figure 7A**), and only dendritic cell infiltration levels affected the survival rate of glioblastoma patients (**Supplementary Figure 7B**). These results indicated that the CNV of certain prognostic genes may determine glioblastoma patient survival in part *via* influencing dendritic cell infiltration. Additionally, it is worth mentioning that C1RL, CD244, CENPN, and EIF4A3 displayed different forms of domain mutation (**Supplementary Figure 8**).

### Immunotherapy Might Be Less Efficient on the High-Risk Group, and the Prognostic Model Showed Optimal Prediction Power

**Figure 8:**
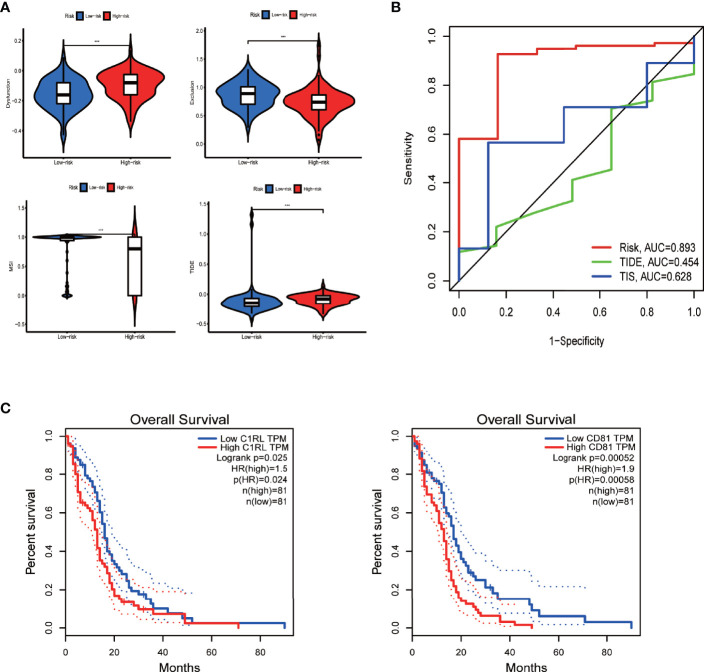
Efficacy prediction of immunotherapy, model comparison, and survival analysis of model genes *via* GEPIA. **(A)** Violin plots showing the TIDE-score analysis of different risk groups in the TIDE database. **(B)** Three-year ROC curve of different prognostic models based on the pROC package. **(C)** Survival curves of model genes significantly associated with survival in GEPIA. ***p < 0.001, **p < 0.01 *p < 0.05, ns: *p* > 0.05.

By analyzing the immunotherapy biomarkers in the high- and low-risk groups, we found higher TIDE scores, lower MSI scores, higher dysfunction scores, and lower exclusion scores in the high-risk group, indicating a greater potential for immune escape and worse immunotherapy efficacy (**Figure 8A**).

Moreover, the performance comparison of different scoring methods showed that the prognostic model we built had a high AUC value of 3-year ROC curve, indicating its greater prediction power than the TIDE and TIS scores (**Figure 8B**).

### Downregulation of CD81 Inhibits Proliferation and Migration and Facilitates Apoptosis in Glioblastoma Cells

**Figure 9:**
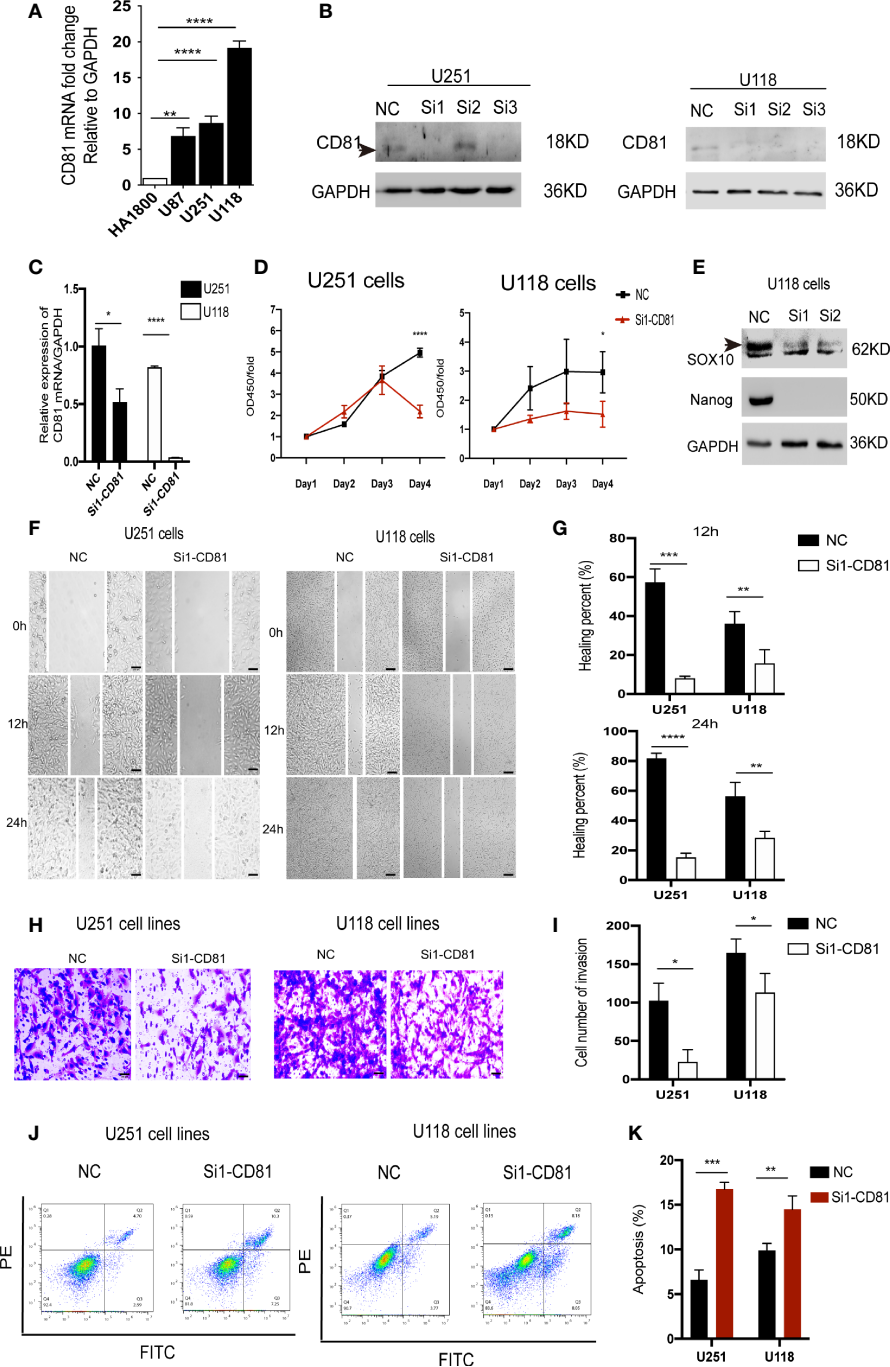
Biological function of CD81 in glioblastoma cells. **(A)** The relative CD81 mRNA levels in glioblastoma and HA1800 cells were measured by qRT-PCR. **(B)** Western blot analysis confirmed that CD81 siRNA inhibited CD81 expression. NC-treated cells served as controls. **(C)** The relative CD81 mRNA expression levels measured by qRT-PCR in NC-treated and Si1-CD81-treated glioblastoma cells. **(D)** The CCK-8 assay was used to detect the effect of CD81 knockdown on the proliferation of U251 and U118 cells. **(E)** Western blot analysis of SOX10 and Nanog in NC-treated and Si1-CD81-treated U118 cells suggested a role of CD81 in stemness regulation of glioblastoma cells. **(F, G)** Representative images and quantitative analysis of the wound healing assay recorded at 0, 12, and 24 h. **(H, I)** Representative images of the transwell assay. Cell staining results and migration cell quantification are shown. **(J, K)** Apoptosis detection using an Annexin V-FITC/PI apoptosis detection kit. ****p < 0.0001, ***p < 0.001, **p < 0.01 *p < 0.05.

Next, we further investigated the biological role of the model genes in glioblastoma. We selected CD81—one of the two genes independently associated with overall survival in glioblastoma patients according to the Gene Expression Profiling Interactive Analysis (GEPIA) server (**Figure 8C**)—for subsequent functional validation (the P of CD81 is smaller). First, qRT-PCR confirmed that glioblastoma cells express higher CD81 levels than astrocytes (**Figure 9A**). The target sequence of each siRNA (Si1, Si2, and Si3) has been uploaded as **Supplementary File 9**. Second, Western blotting (**Figure 9B**) and qRT-PCR (**Figure 9C**) confirmed the gene silencing effect of siRNA for CD81. Based on these results, we used Si1 for both U251 and U118 cells in the subsequent assays (the knockdown effect was unconvincing only in Si2 for U251 cells, and this did not affect subsequent experiments). The CCK8 assay indicated that knocking down CD81 significantly inhibited glioblastoma cell proliferation (**Figure 9D**). The Western blot analysis of SOX10 and Nanog in NC-treated and Si-CD81-treated U118 cells indicated that knocking down CD81 weakened the stemness of glioblastoma cells (**Figure 9E**). The wound healing assay and transwell invasion assay revealed that CD81 inhibition markedly inhibited the migration of U251 and U118 cells (**Figures 9F–I**.). Next, flow cytometry confirmed that knocking down CD81 promoted apoptosis in U251 and U118 cells (**Figures 9J, K**). These results suggest that knocking down CD81 suppressed proliferation and migration and facilitated apoptosis in U251 and U118 cells. Additionally, knocking down CD81 significantly reduced the stemness of U118 cells, which calls for further in-depth studies.

## Discussion

Discovery and integrative analysis of prognostic biomarkers help clinicians refine treatment and predict disease outcomes. Furthermore, in-depth studies could reveal related pathophysiological mechanisms. By applying multiple bioinformatics algorithms, we successfully developed a high-performance prognostic model based on m6A-associated immune genes. We also developed and published a corresponding web version of the dynamic nomogram application, enhancing utility and translational meaning. Moreover, this study provided and assessed different glioblastoma patients stratification methods and explored the correlation between different methods. Finally, a multi-omics analysis of model genes enriched the research content.

Biomedical big data research provides unprecedented opportunities for biomedical development, bringing many new technologies and methods for disease diagnosis and treatment, such as gene diagnosis, gene therapy, and targeted drugs. Regarding glioblastoma, various prognostic models now exist (43–45). We developed the first high-performance prognostic model based on m6A-associated immune genes; it successfully passed internal and external validation tests and has some clinical translation value.

Public databases now contain numerous similar studies about the model genes involved in this study. Silencing the DEK gene in U251 glioblastoma cells inhibited cell proliferation and induced cell apoptosis by upregulating tumor suppressor genes (P53 and P21) and downregulating oncogenes (Bcl-2 and C-myc) (46). In another study, Wang et al. (47) observed that pIRESne3-CMIP transfection dramatically increased proliferation and metastasis in U251 glioma cells with low CMIP expression levels. A recent study (48) confirmed that EIF4A3 played a role in the EIF4A3/CASC2/RORA loop and ultimately facilitated the aggressive phenotype of glioblastoma. Moreover, EIF4A3 induced the formation of circular RNA ASAP1 (49) and MMP9 (50), which both promoted glioblastoma tumorigenesis. In a study investigating immune system-related plasma proteins in glioblastoma (51), researchers found that high CD244 levels were associated with long progression-free survival, which seems contradictory to our results. An *in vitro* experiment by Wu et al. (52) proved that CENPN expression levels were positively associated with the WHO grade of glioma and that CENPN promoted malignant glioma cell phenotypes. Although OGFOD1, C1RL, CD81, and ITPKC play pivotal roles in several cancers, their involvement in glioblastoma remains undocumented (53–56).

The single-sample GSEA algorithm and clustering grouping indicated that the high-m6A score group corresponded to the groups with medium and low m6A-regulating gene expression and the group with high m6A-related immune genes, with lower survival rates and a higher degree of immune infiltration. Multi-GSEA enrichment results provided a possible explanation: a higher immune infiltration in the TME with enhanced immunosuppressive activity, involving various immune cell biological behaviors and multiple signaling pathways and eventually lowering the survival rate of glioblastoma patients. Some bioinformatic analyses indicated that m6A regulators were closely related to immune infiltration and immunotherapy efficacy (44, 57, 58). A recent study by Dong et al. showed that hypoxic conditions induced expression of the “eraser” ALKBH5 in glioblastoma models, facilitating immunosuppression through an epitranscriptomic mechanism (59). Another study found that m6A-regulated long non-coding RNA HSPA7 facilitated macrophage infiltration through the YAP1–LOX axis and enhanced the efficiency of anti-PD1 therapy in glioblastoma (60). However, the concrete mechanism by which m6A regulators affect immune response in glioblastoma remains obscure and requires further studies.

Combined with the coexpression network, the above results indicated broad interactions between m6A-regulated and immune genes in glioblastoma. These genes were closely related to immune activity suppression and therefore had important biological significance.

Moreover, the mutation burden further refined the stratification of the survival rate of glioblastoma patients in the high- and low-risk groups, leading to more accurate prognoses. The multi-omics analysis of model genes suggested that the expression levels of some model genes and CNV also affected immune cell infiltration in glioblastoma, reflecting the comprehensiveness and complexity of regulatory factors in the TME. After identification from GEPIA, we chose CD81 for further investigation. It was worth mentioning that the coefficient alone was an inappropriate index to measure the contribution of model genes, as the expression level of CD244 was prominently lower than other model genes (Raw Data-TCGA-symbol.xlsx). Additionally, an existing study has shown the role of CD81 in mediating radioresistance in glioblastoma cells (61), and our study may form a good complement to this study. In a word, positive results in different databases and the existing research foundation were the reasons why we choose CD81 as the subject.

We observed that interfering with CD81 expression inhibited proliferation, migration, and invasion and promoted apoptosis in U251 and U118 glioblastoma cells *in vitro*, suggesting a potential role of CD81 in glioblastoma diagnosis and prognosis. However, the experimental validation result (**Figure 9E**) contradicted the predicted relationship between CD81 expression and cell stemness (**Supplementary Figure 5**), suggesting a limitation of our bioinformatics analysis. Another limitation of our study was that we did not have enough energy and layout to examine the effect of CD81 knockdown on immune regulation of glioblastoma, which may provide inspiration for further studies.

## Conclusions

In summary, we constructed a high-efficacy prognostic model for glioblastoma patients, and the dynamic nomogram and immunotherapy efficacy prediction web application enhanced clinical translational significance. Additionally, the correlation of the two clusters and m6A score with the TME revealed the pathophysiological process of glioblastoma. Finally, our *in vitro* experiments suggested the significance of CD81, one of the model genes, as a diagnostic and prognostic biomarker. Therefore, this study provides tools for accurate glioblastoma prognosis and ideas and inspiration for in-depth mechanism studies.

## Data Availability Statement

The original contributions presented in the study are included in the article/**Supplementary Material**. Further inquiries can be directed to the corresponding authors.

## Ethics Statement

Written informed consent was obtained from the individual(s) for the publication of any potentially identifiable images or data included in this article.

## Author Contributions

GH, XP, and YZ conceived and designed the study. NL, XS, MF, and XL performed the literature search, generated the figures and tables, and wrote the manuscript. SM, WZ, QL, and FY supervised the study and reviewed the manuscript. All authors read and approved the final manuscript.

## Funding

This study was funded by the National Natural Sciences Foundation of China (Grant Nos. 82003312 and 82173311).

## Conflict of Interest

The authors declare that the research was conducted in the absence of any commercial or financial relationships that could be construed as a potential conflict of interest.

## Publisher’s Note

All claims expressed in this article are solely those of the authors and do not necessarily represent those of their affiliated organizations, or those of the publisher, the editors and the reviewers. Any product that may be evaluated in this article, or claim that may be made by its manufacturer, is not guaranteed or endorsed by the publisher.

## Supplementary Material

Supplementary Figure 1 Kaplan-Meier survival analysis curves of glioblastoma patients grouped by m6A-regulating genes levels with P < 0.05. For all these genes, the high- and low-expressing groups had significantly different survival curves.

Supplementary Figure 2 Forest plots of univariate and multivariate COX regression analyses. **(A)** Univariate COX regression analysis indicated that age and risk score were two prognostic factors of glioblastoma patients. **(B)** Multivariate COX regression analysis demonstrated that risk score determined glioblastoma outcome independently of age and sex.

Supplementary Figure 3 Mutation analysis of different risk groups. **(A)** Mutational waterfall diagram of the high-risk group. The colors represent different mutation patterns. **(B)** Mutational waterfall diagram of the low-risk group. The right side of the plot indicates gene mutation frequency. **(C)** Survival analysis of the high and low mutation burdens combined with the risk score.

Supplementary Figure 4 The choice of the number of clusters (K value). Both **(A)** and **(B)** display the corresponding growth of the area under the cumulative distribution function curve and the tight intra-group linkage. **(A)** K =3 is appropriate for the clustering based on m6A-regulating gene expression. **(B)** K =3 is appropriate for the clustering based on prognostic m6A-associated immune gene expression.

Supplementary Figure 5 Statistical analysis of the correlation between model genes and stemness and TME scores in glioblastoma patients. Stemness scores based on DNA methylation and RNA in UCSC Xena database are shown. TME scores include stromal score, immune score, and estimate score.

Supplementary Figure 6 Variation frequency of model genes. **(A)** CNV frequency of model genes in TCGA-glioblastoma samples based on TIMER database. **(B)** Mutation frequency of model genes in TCGA-glioblastoma samples based on cBioportal database.

Supplementary Figure 7 Influence of model genes’ CNV. **(A)** Statistical analysis of the correlation between the CNV of model genes and immune cell infiltration levels in glioblastoma based on TIMER database. **(B)** Effect of immune-cell infiltration on the survival rate of glioblastoma patients based on TIMER database. ***P < 0.001, **P < 0.01 *P < 0.05.

Supplementary Figure 8 Domain mutations of model genes. The red and green squares indicate different domains, and the lines and dots above indicate nonsense mutation sites.
