# Supplementary material for: Development of a Novel Prognostic Model of Glioblastoma Based on m6A-Associated Immune Genes and Identification of a New Biomarker
Source: Front Oncol. 2022 Jul 20;12:868415. doi: 10.3389/fonc.2022.868415 (PMC9348864; doi:10.3389/fonc.2022.868415)
Supplement: Supplementary file 1 [file DataSheet_1.docx]

Raw data of bioinformatic analysis and in vitro study were uploaded in:

<https://www.jianguoyun.com/p/DcRUngMQ0KafChjeq6wEIAA>
