# Supplementary material for: Development of a Novel Prognostic Model of Glioblastoma Based on m6A-Associated Immune Genes and Identification of a New Biomarker
Source: Front Oncol. 2022 Jul 20;12:868415. doi: 10.3389/fonc.2022.868415 (PMC9348864; doi:10.3389/fonc.2022.868415)

Supplementary Figure 1

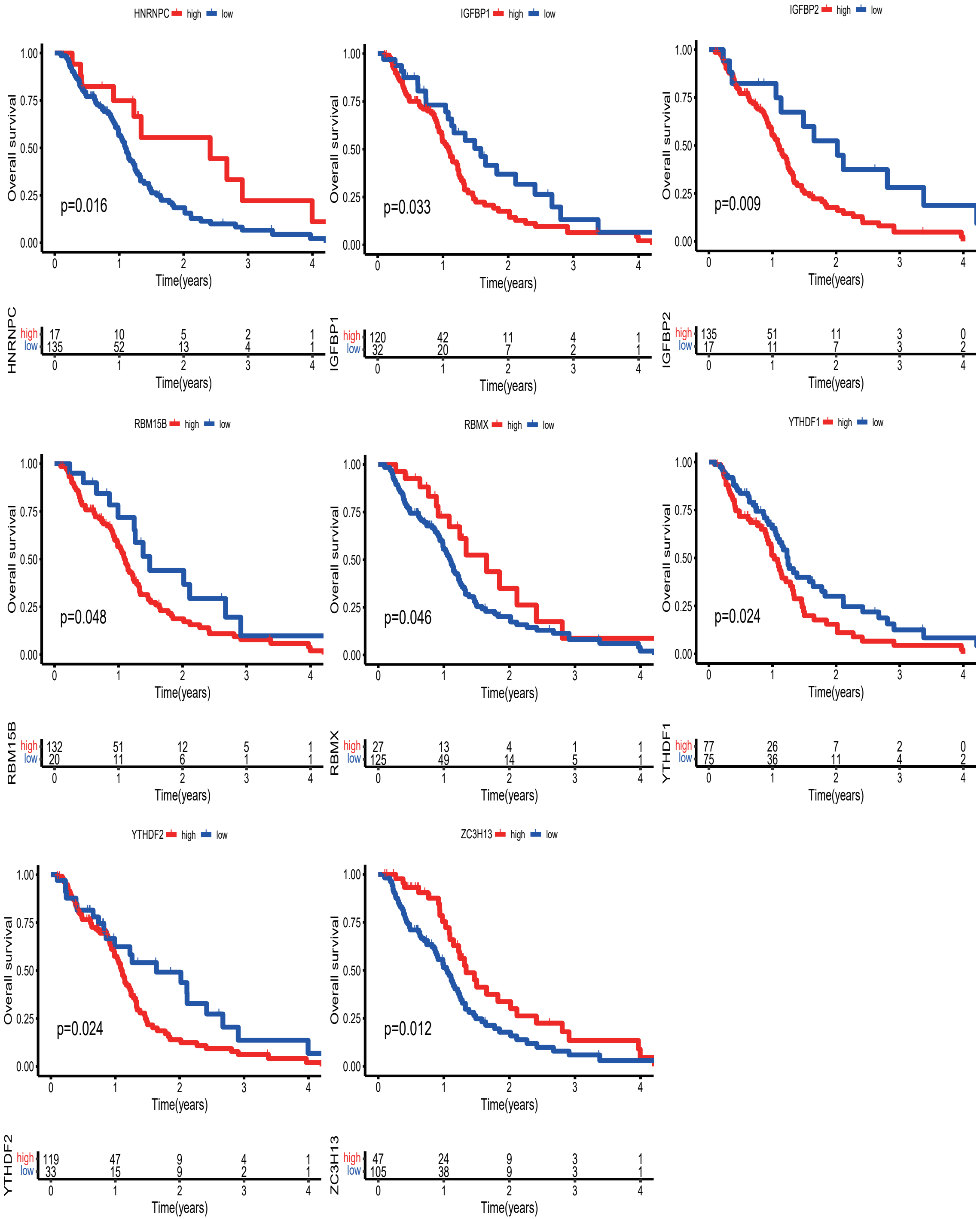

Supplementary Figure 2

A

	pvalue	Hazard ratio
age	<0.001	1.035(1.017−1.053)
gender	0.338	0.824(0.555−1.224)
IDH	0.065	0.389(0.143−1.060)
G−CIMP	0.006	0.195(0.061−0.618)
riskScore	0.003	1.003(1.001−1.006)

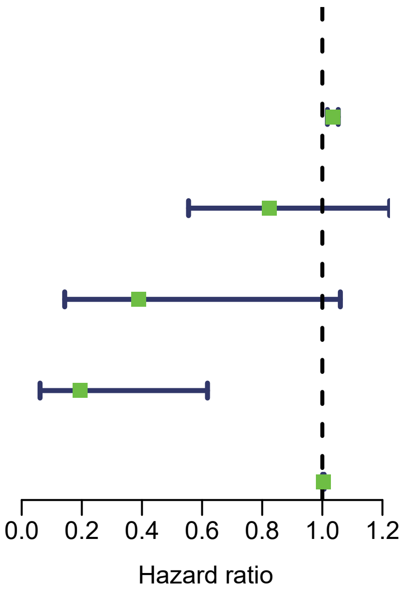

B

	pvalue	Hazard ratio
age	0.002	1.031(1.012−1.050)
gender	0.929	0.982(0.655−1.472)
IDH	0.093	3.429(0.815−14.427)
G−CIMP	0.014	0.131(0.026−0.659)
riskScore	0.007	1.003(1.001−1.005)

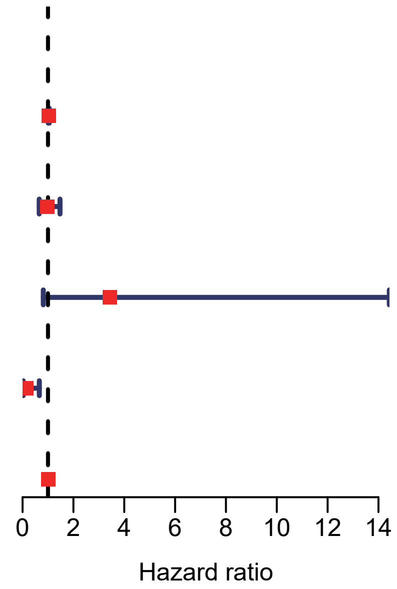

Supplementary Figure 3

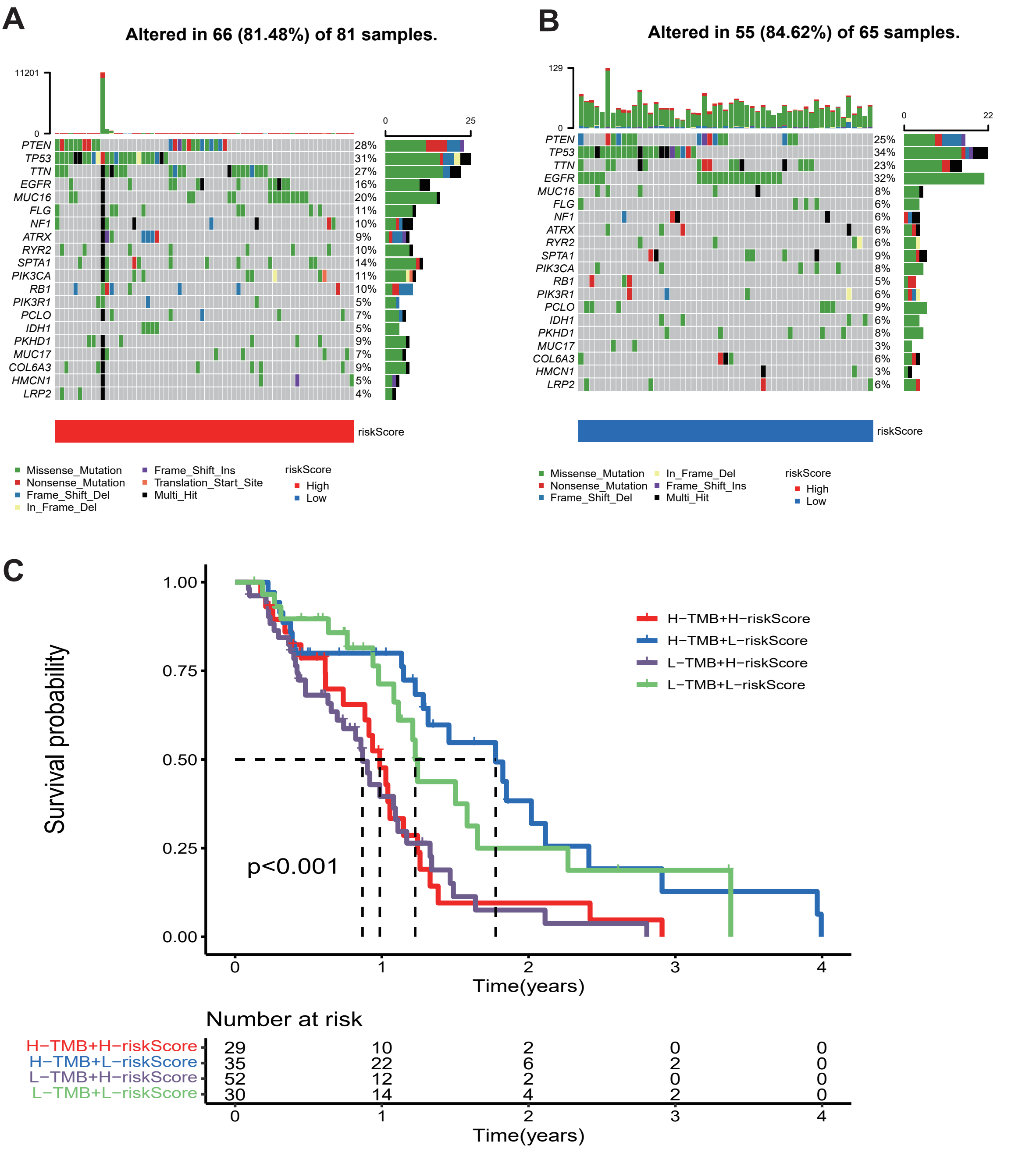

Supplementary Figure 4

A

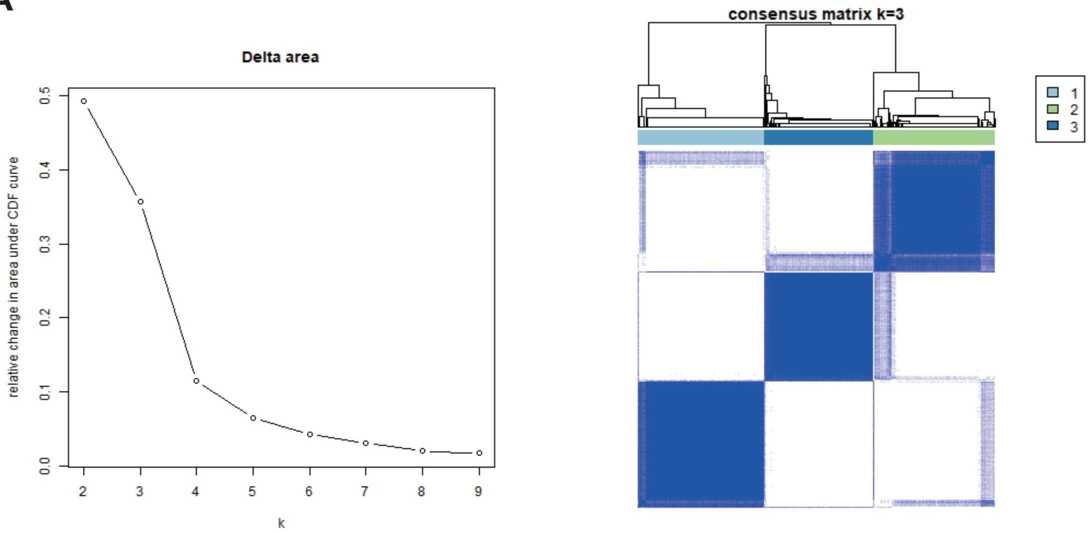

B

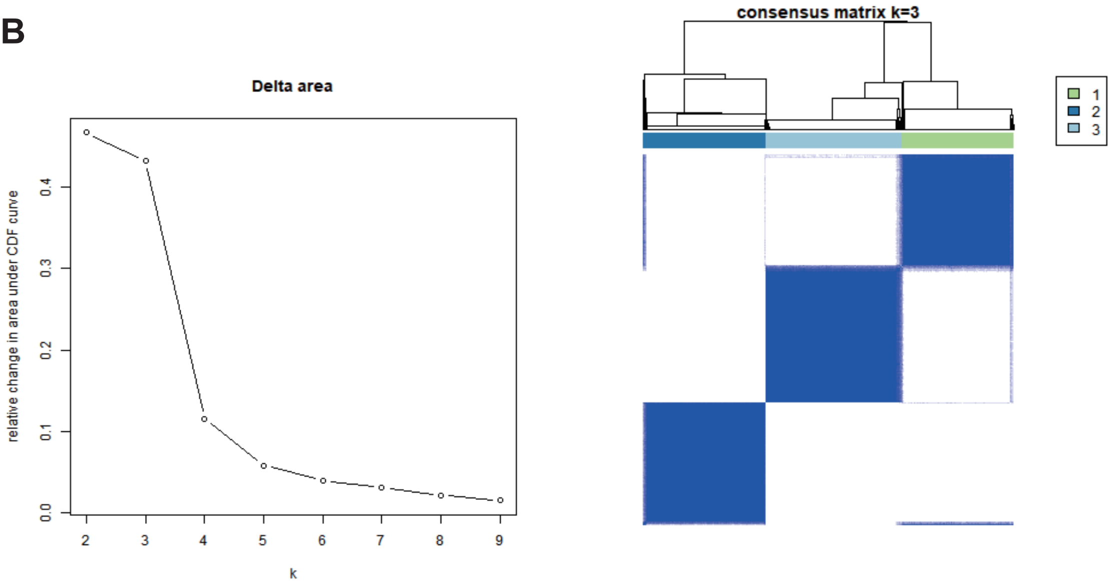

Supplementary Figure 5

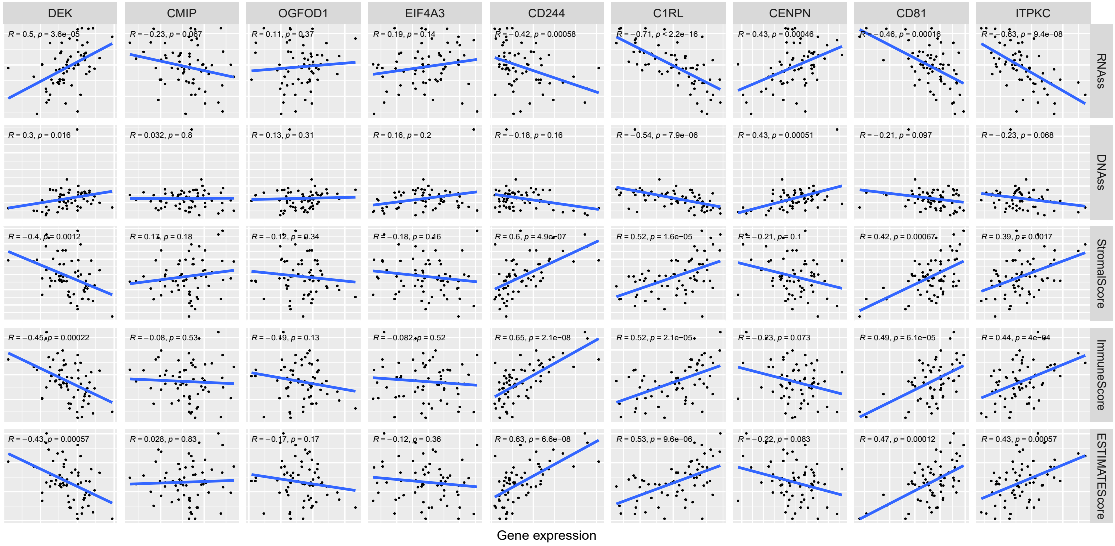

Supplementary Figure 6

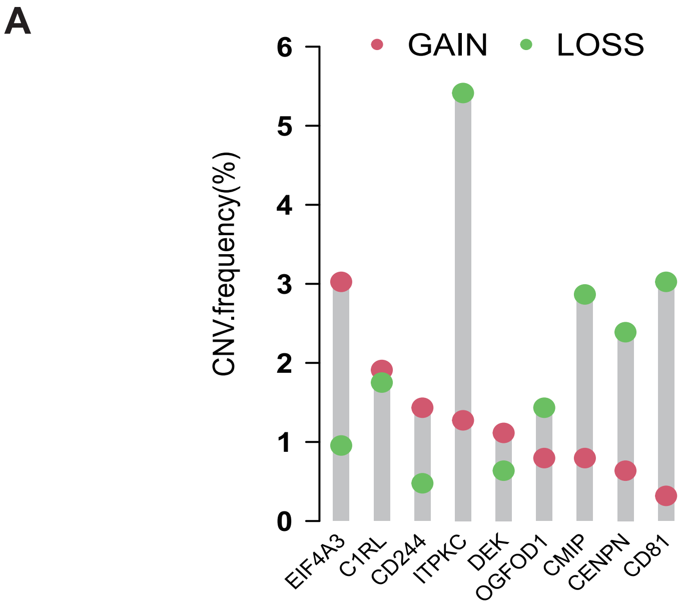

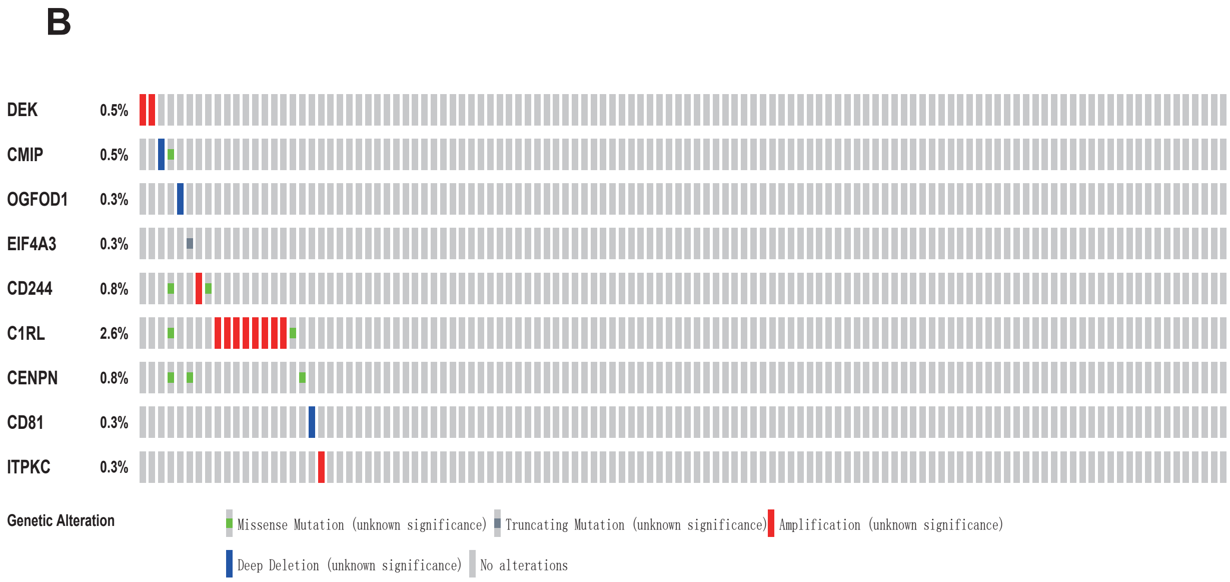

Supplementary Figure 7

A

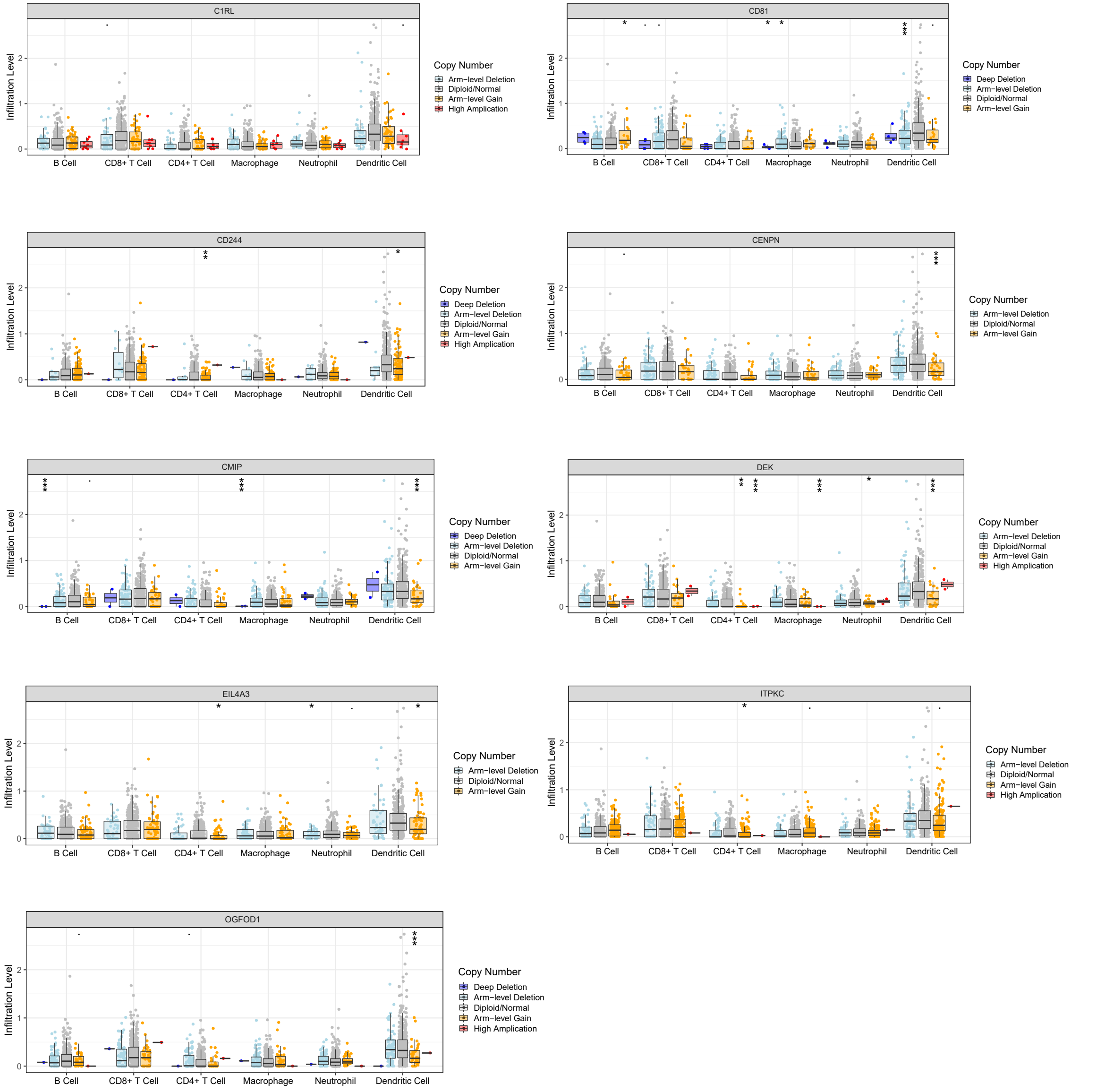

B

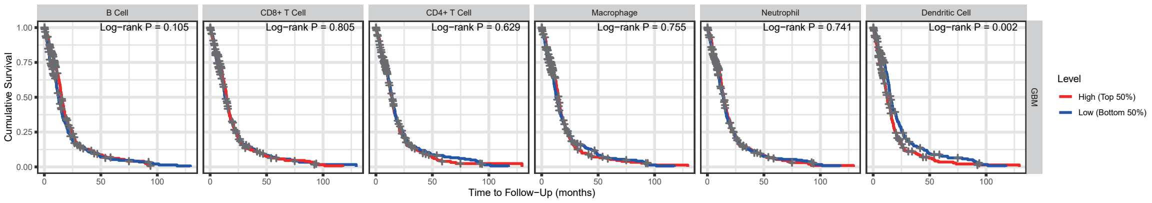

Supplementary Figure 8

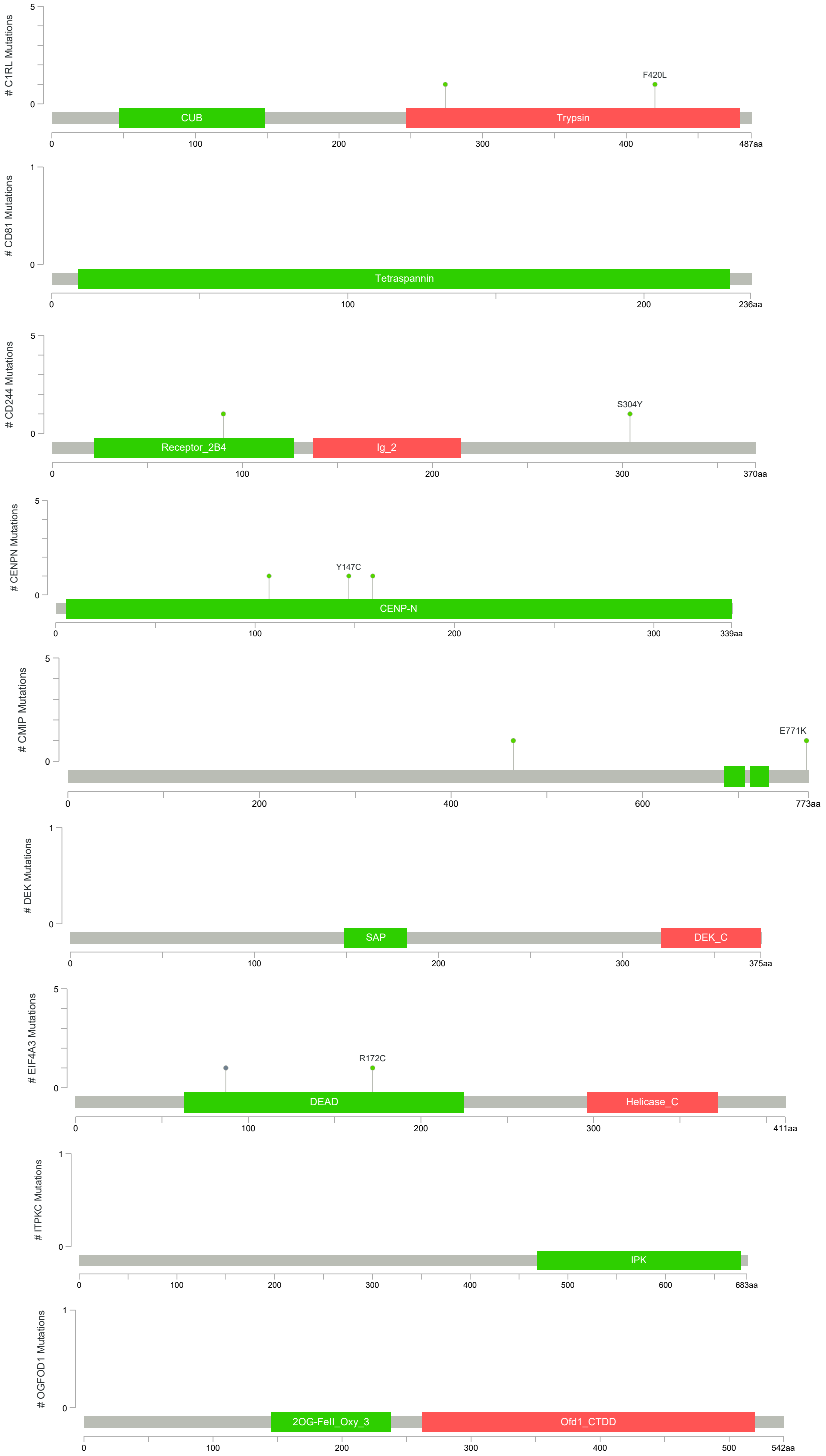

Supplement: Supplementary Figure 1 — Kaplan-Meier survival analysis curves of glioblastoma patients grouped by m6A-regulating genes levels with P < 0.05. For all these genes, the high- and low-expressing groups had significantly different survival curves. [file DataSheet_2.pdf]
